# Bacteria autoaggregation: how and why bacteria stick together

**DOI:** 10.1042/BST20200718

**Published:** 2021-06-10

**Authors:** El-shama Q. A. Nwoko, Iruka N. Okeke

**Affiliations:** Department of Pharmaceutical Microbiology, Faculty of Pharmacy, University of Ibadan, Ibadan, Oyo State, Nigeria

**Keywords:** adherence, autoaggregation, biofilm, self-associating

## Abstract

Autoaggregation, adherence between identical bacterial cells, is important for colonization, kin and kind recognition, and survival of bacteria. It is directly mediated by specific interactions between proteins or organelles on the surfaces of interacting cells or indirectly by the presence of secreted macromolecules such as eDNA and exopolysaccharides. Some autoaggregation effectors are self-associating and present interesting paradigms for protein interaction. Autoaggregation can be beneficial or deleterious at specific times and niches. It is, therefore, typically regulated through transcriptional or post-transcriptional mechanisms or epigenetically by phase variation. Autoaggregation can contribute to bacterial adherence, biofilm formation or other higher-level functions. However, autoaggregation is only required for these phenotypes in some bacteria. Thus, autoaggregation should be detected, studied and measured independently using both qualitative and quantitative *in vitro* and *ex vivo* methods. If better understood, autoaggregation holds the potential for the discovery of new therapeutic targets that could be cost-effectively exploited.

## Introduction

Bacteria often exist in consortia, adhering either to surfaces, non-bacterial cells, or other bacteria. Bacterium-bacterium adhesion of genetically identical strains is referred to as autoaggregation, while inter-strain adherence of genetically distinct strains, of the same or different species, is co-aggregation. While we acknowledge that the mechanisms and contributing factors for both auto- and co-aggregation overlap, this review will focus largely on autoaggregation, which may be less common in nature but is better understood microbiologically. Excellent recent reviews on auto- and co-aggregation have been recently published elsewhere [1–6]. Aggregation occurs due to chemical or electrostatic interaction between cell surface molecules, which can self-associate or bind a distinct and different receptor.

## Autoaggregation effectors

### Fimbriae

**Figure 1.**
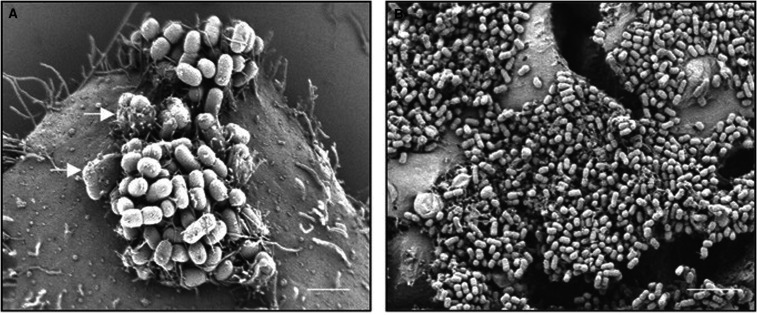
Localized adherence of typical enteropathogenic *Escherichia coli* (EPEC E2348/69) on HT-29 cells is the formation of adherent autoaggregants mediated by bundle-forming pili and stabilized by other adhesins [8]. (**A**) The formation of adherent autoaggregants mediated by bundle-forming pili At 3 h postinfection of HT-29 cells with EPEC E2348/69) (**B**) this property allows for dense and efficient colonization of epithileal cells within 6 h. Saldana et al. 2009 [8].

Fimbriae or pili are structural organelles that confer a range of functions on bacteria, many of them adhesive. The enteropathogenic *Escherichia coli* bundle-forming pili are autoaggregation effectors that mediate localized adherence on epithelial cells, resulting in microcolonies, which are tightened by pilus retraction and stabilized by other adhesins such as the *Escherichia* common pilus (Figure 1) [7, 8]. Other type IV fimbriae mediating autoaggregation include the many types of enteroaggregative *E. coli* aggregative adherence fimbriae [9–12] and *Neisseria meningitidis* type IV pili [13, 14]. These retractable pili typically mediate host cell adherence by the binding of pilus tip proteins to specific receptors and autoaggregation by lateral, bundling interactions among the main structural subunits of different pili [13]. These dual function adhesins are important in the initiation, growth, maintenance and disassembly of auto-aggregates (microcolonies) within infection niches. Further examples of surface organelles involved in autoaggregation are the *Vibrio cholerae* DNA-uptake pili [15], curli [16], *Edwardsiella piscida* EseB filaments [17] and the *Vibro vulnificicus* Tad pili [18].

### Afimbrial adhesins

Prominent among Enterobacteriales adhesins are the type V secreted proteins, otherwise referred to as autotransporters. These include, but are not limited to, the *Veillonella* monomeric autotransporters A-C [19]; the Serine Protease Autotransporters of Enterobacteriaceae (SPATEs) such as the tandem autotransporter B and C (TagB and TagC), the serine-protease hemagglutinin autotransporter *sha* [20]; the *E. coli* Antigen 43 (Ag43 or Flu) [21] and trimeric autotransporter adhesins such as EibC, EibD, *Yersinia* adhesin A (YadA) of enteropathogenic *Yersiniae enterocolitica* (YeYadA) and *Y. pseudotuberculosis* (YpYadA) [22] and the *Veillonella* trimeric autotransporters A-I [19]. Integral β-barrel proteins can also confer autoaggregation [23].

Adhesins may bind to heterologous receptors but autoaggregation is often achieved through self-association. The *N. meningitidis* PilE pilus structural subunit mediates autoaggregation by electrostatic means, requiring a C-terminal lysine residue [13]. In contrast, the heat-resistant agglutinin 1 (Hra1) and its allelic variant Hek [24] depend on specific interaction motifs, but their structural basis for self-association is presently unknown [25]. Crystal structures for a few self-associating autotransporters (SAATs) are available and provide the best insight into self-association mechanisms.

**Figure 2.**
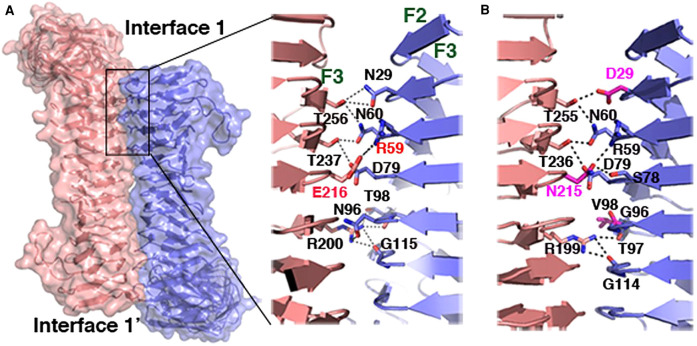
Velcro-like self-association between identical L-shaped passenger domains of adjacent *Escherichia coli* Ag43 molecules [26]. The interaction is held by nine hydrogen bonds [N29–T256 (two hydrogen bonds), N60–T256, N60–T237, D79–T237, N96–R200, T97–R200, T98–R200, G115–R200] and a salt bridge between the R59 and E216 side chains. Reproduced from Heras et al. 2014 [26] with permission.

**Figure 3.**
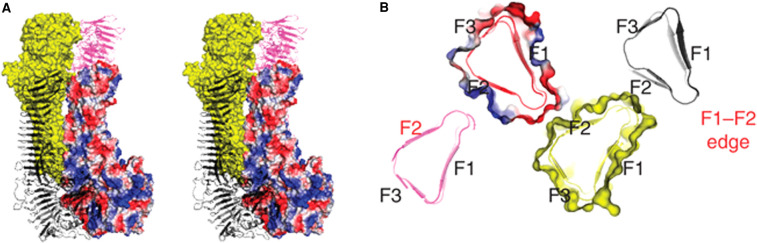
Self association between components of a *Haemophilus influenzae* multimer. (**A**) Interactions among four Hap molecules shown in surface (coloured in yellow and by electrostatic surface potential) and cartoon (black and magenta) representations, respectively. (**B**) Slab view of the packing interface of the Hap–Hap multimer at a cross-section in the primary interaction site of D776–N777. The F1/F2/F3 faces are labelled. The F2 face and F1–F2 edge at the growing ends of the multimer are highlighted in red. Reproduced from Meng et al. 2011 [27] with permission.

Antigen 43 self-associates via a velcro-like mechanism (Figure 2) [21, 26]. The interacting interfaces are on this SAAT's L-shaped passenger domain comprising a stem (SL), an elbow (EJ) and the bottom (BL) subdomains. Each of these three subdomains has two subtypes: SL2 and SL2; EJ1 and EJ2; and BL1 and BL2 and evolutionary shuffling of these passenger subdomains subtypes gives rise to four distinct Ag43 passenger subclasses. All passenger subtypes preferentially autoaggregate and heterotypic associations occur majorly between subclasses having the same SL subtype [26]. The self-associating passenger domain of the *Haemophilus influenzae* Hap protein, another SAAT, folds into a three-face prism with hydrophilic residues on the outside and a hydrophobic core. One face of each prism, the F2 face interacts with the F1–F2 edge of another, forming a multimeric lattice (Figure 3) [27]. Other SAATs do not share features of either Ag43 or Hap; it is highly likely that other self-associating mechanisms exist and that these functions evolved convergently.

### Secreted macromolecules

Autoaggregation can be the indirect consequence of a secreted factor mediating aggregation indirectly by connecting other surface factors [28]. Proteinase K treatment more commonly alters or completely obliterates autoaggregation compared with sodium periodate (polysaccharide removal) and DNase1 (extracellular DNA removal) treatment [29–31]. However, polysaccharides as well extracellular DNA, released during autolysis can mediate autoaggregation. Myrtenol, a bioactive plant derivative that inhibits autolysis, produces a significant decrease in the autoaggregation ability of methicillin-resistant *Staphylococcus aureus* [32].

## Modulation of autoaggregation

Calcium induces autoaggregation in *Aeromonas hydrophila* culture [30] when it is the dominant exchangeable ion in the medium [16]. This effect can be produced by gallic acid in *Actinomyces naeslundii* culture [33], and sodium chloride in *Pediococcus pentosaceus* R1 *and Lactobacillus fermentum* R6 cultures [28, 34]. These examples illustrate that autoaggregation is a regulated and environmentally responsive phenotype.

Regulation can be transcriptional, post-transcriptional or even arise from interactions among different surface factors [35]. Adhesins, particularly short ones like integral outer membrane proteins and autotransporters can be masked or shielded by exopolysaccharides (EPS), fimbriae and even secreted proteins [23, 35–38, 39 19]. In *Lacticaseibacillus rhamnosus*, EPS production masked adherence by *spaCBA* pili [38] and encapsulated *Pasteurella multocida* cells autoaggregated less than non-encapsulated (capsule-deficient) *P. multocida* [40]. Capsule interference could be due to its electrostatic negative charge [40] or sterical obstruction of autoaggregation factors [41]. Autoaggregation is therefore the product of complex surface-factor choreography, which ensures that autoaggregation factors are masked and unmasked when necessary; and it is carefully regulated transcriptionally and post-transcriptionally.

### Transcriptional regulation

The cellular levels of the ubiquitous bacterial second-messenger cyclic dimeric guanosine monophosphate (c-di-GMP), involved in planktonic/sessile transitions and EPS production, is regulated by phosphodiesterase (*pde*) degradation. In *Erwinia amylovora,* deletion of *pdeABC* resulted in the formation of well-defined aggregates with increased amylovoran, an exopolysaccharide, and cellulose production [42]. In Gram-negative bacteria, envelope stress from environmental changes is detected and responded to by the two-component signal/regulatory transduction system CpxA/CpxR. Environmental cues cause CpxA to autophosphorylate and then phosphorylate the cytoplasmic response regulator, CpxR. CpxR increases adherence and autoaggregation but attenuates virulence. Deletion of CpxR results in reduced autoaggregation and biofilm formation in *Proteus mirabilis* and *Salmonella enteritidis* but increased expression of essential *Salmonella* virulence genes [43, 44].

### Epigenetic regulation

Many autoaggregation factors are regulated epigenetically through phase variation and Antigen 43 is the textbook example. *E. coli* that have Ag43 in Phase-ON produce rough colonies and very obvious clumping in liquid cultures [45]. Deoxyadenosine methylase (Dam) methylates GATC sites upstream of the Antigen 43 gene (*agn43*, *flu*); when unmethylated, these sites are occlusively bound by the global oxidative stress response protein, OxyR, which represses transcription*. E. coli dam* (deoxyadenosine methylase) mutants are locked in Phase-OFF while *oxyR* mutants are locked in Phase-ON. These and other findings support a model in which methylation of DNA upstream of the *agn43* promotor prevents repressor OxyR binding. At replication, if OxyR binds to the promoter before Dam methylates the GATC sites, the progeny of Ag43-expressing cells become locked in Phase-OFF mode, and therefore produce smooth, non-aggregating strains in which Ag43 cannot be detected [46].

Phase variation permits strains with strongly self-associating proteins to switch them off in a sub-population at times or in niches where autoaggregation may be deleterious. The resultant population heterogeneity is evolutionarily advantageous for the lineage. As has been demonstrated with Antigen 43, autoaggregation is often an asset for initializing colonization but can get in the way of maintaining colonization or invasion [47]. Thus phasing of autoaggregation is evolutionarily advantageous to persistent colonizers. The Hag/MID —Haemagglutinin/*Moraxella* IgD binding protein— is an autotransporer protein detectable in *Moraxella catarrhalis* isolates from newly infected patients but not expressed by *M. catarrhalis* isolates from chronic obstructive pulmonary disease, which have reduced autoaggregation. Switching in this case is by slipped-strand mispairing [48].

### Post-translational regulation

Epigenetic control results in permanent ON or OFF status in individual cells until cell division permits a phase change. Phase-ON bacterial cells bound in autoaggregates cannot therefore exit, unless dislodged by shear force or by proteolysis. The *H. influenzae* Hap has a proteolytic domain separate from its self-associating domain that effectively cleaves the molecule off the cell surface [49] offering a ‘built in’ escape mechanism. Escape mechanisms are under-investigated and likely common-place as they overcome autoaggregation when it poses a selective disadvantage. After autoaggregation and adherence have been established by *N. meningitidis*, the bacterium adds a phosphoglycerol moiety to its type IV pili. With this modification the pili continue to adhere but no longer self-associates, thereby releasing bacteria not in contact with host cells to seed other infection foci [50]. Autoaggregation effectors can also be countered by antiaggregation proteins. Deletiing the gene encoding the enteroaggregative *E. coli* anti-aggregation protein (Aap), also known as dispersin, produces exaggerated clumps of these highly aggregative bacteria [51]. Aap was initially believed to electrostatically optimize the placement of fimbriae around bacterial cells, and may indeed function this way in part [52]. However, secreted Aap on the cell surface [53], effectively masks the integral outer-membrane autoagglutinin Hra1. *Aap* and *hra1* double mutants are non-autoaggregating [35].

## Laying bare confounding: autoaggregation-associated phenotypes

Biofilm formation is a common sequel of aggregation [54] but the phenotypes are seperable. In instances where aggregates —and even biofilms— detach or float, a relationship between autoaggregation and biofilm mass may not be seen in conventional laboratory assays that measure biofilms on horizontal solid supports but may be more visible on vertically mounted surfaces [35]. Biofilms on horizontal surfaces benefit from gravitational pull on planktonic cells and biofilm aggregates while autoaggregation is less critical to shear stress-mediated biofilm formation in continuous flow systems [55]. In static culture, autoaggregation and biofilm of *V. parvula* SKV38 formation correlate but in dynamic flow, a non-aggregating *Veillonella* trimeric autotransporter gene mutant formed six times more biofilm than the wildtype. Biofilm formation of *Erwinia amylovora* is similarly increased by continuous flow compared with static culture likely because shear stress mediates *E. amylovora* biofilm in the plant xylem [42].

Motility is an important contributor to biofilm formation that is not required for autoaggregation. *Salmonella* Enteritidis *cpxR* mutants show increased swimming motility but reduced autoaggregation, biofilm mass and expression of adhesion-related genes [44]. In *Pseudomonas aeruginosa* PAO1, flagellar motility appears to be necessary for surface attachment (biofilm) but not autoaggregation [56]. Cell surface hydrophobicity can correlate with adherence or autoaggregation but the correlative phenotype arises from the physicochemical properties of individual adhesins. In some studies, a reduction in hydrophobicity increased autoaggregation [34, 57–59] but reduced cell charge [58], adherence [33] and biofilm mass [60].

Altogether, although many phenotypes are associated with autoaggregation. When assayed, autoaggregation should be verified microscopically or measured indirectly but specifically through settling assays to avoid confounding [61].

## Evolutionary benefits of autoaggregation to bacteria

### Niche/host colonization

Autoaggregation can be important for niche entry, establishment and maintenance in host colonization and disease pathogenesis. The immunogenic *L. pneumophila* collagen-like protein, Lcl, mediates both autoaggregation for niche colonization and cell to surface attachments in biofilms. It has variable numbers of an immunogenic tandem collagen-like gly-Xaa-Yaa (GXY) repeat. Autoaggregation and biofilm formation increase with GXY repeat numbers; thus, *L. pneumophila* strains with more than 18 GXY tandem repeats are rarely implicated in clinical cases for two probable reasons: they are hardly aerosolized, and therefore not droplet-transmissible, due to their hard-to-dislodge and tightly packed environmental biofilm, or they are highly antigenic and thus, are cleared by the immune system [62].

### Kin recognition

Co-aggregating strains preferentially aggregate with other strains displaying the same or similar surface adhesins. Similarities and differences among adhesins, or alleles of the same adhesin, serve as discrimination cues for preferential kin/self-interaction (autoaggregation) or mixed interaction (coaggregation) between strains [15]. Autoaggregation mediated kin-recognition has been observed in *Vibro cholerae* strains via specific preferential PilA–PilA interaction [15] and *Escherichia coli* strains via specific immunoglobulin-binding Eib autotransporters or Ag43 subclass association [21, 22].

### Kind-recognition

To modulate the greenbeard effect of kin-recognition, *E. coli* strains carry different alleles of Ag43 [21]. Diversity and multiplicity in the carriage of adhesins favour differential and beneficial nepotic kind-interactions, i.e attraction to organisms carrying similar aggregating factor(s) and exclusion of non-kind. An attractive hypothesis is that pathogens evolve adhesins that can recognize and latch onto commensal aggregation factors to enter and establish themselves in a niche. While evidence to fully support this hypothesis remains to be collated, Rck, an integral outer membrane invasin of *Salmonella,* contains an autoaggregation motif present in the *E. coli* (Hra1). Hence, the *E. coli* Hra1 can mediate co-aggregation with *Salmonella* strains expressing Rck, at least *in vitro* [25]. Similarly, Antigen 43 self-associates but can also associate with SAATs of diarrhoeagenic *E. coli* strains such as AIDA-1 and TibA [63].

### Out-competition

During niche colonization, autoaggregation of strains via kin-recognition favours out-competition of discriminated strains. YadA of enteropathogenic *Yersiniae enterocolitica* (YeYadA) and YadA of *Y. pseudotuberculosis* (YpYadA) both have the YadA-like head domain; however, YpYadA has an additional uptake region. In co-culture, YeYadA and YpYadA isogenic mutants preferentially autoaggregated (excluding the other), rather than coaggregated, suggesting that the YpYadA uptake region is a structural discriminating cue for coaggregation (inclusion) between YeYadA and YpYadA mutants strains. This structural difference between their respective YadAs and consequent other-exclusion has been hypothesized to be responsible for the rarity of *Y. enterocolitica* and *Y. pseudotuberculosis* co-infection even in prevalent areas [22].

### Surviving environmental stress

Autoaggregation and biofilm formation confer antimicrobial resistance, metabolic cooperation, virulence factor production and survival and persistence in certain niches or hosts [1]. Compared with planktonic *Pseudomonas aeruginosa,* non-attached *P. aeruginosa* aggregates were tolerant to bactericidal gentamicin and carbenicillin. Conversely, disrupted aggregates showed significantly increased susceptibility to both antibiotics [56].

### Selective disadvantages of autoaggregation in disease pathogenesis

Autoaggregation in the absence of cell to surface adhesion is disadvantageous to pathogenic strains in infection as it can enhance outcompetition and immune clearance [62, 64]. Autoaggregation enhances biofilm formation but overall coverage of surface/substratum is determined by cellular surface attachment [58, 59]. Thus, hyper-autoaggregation resulted in the formation of centred-aggregates and biofilms instead of dispersed-aggregates and spread-out biofilm in wild type *Streptococcus pyogenes* [64]. To circumvent reduced colonization and subsequent out-competition from the niche, some bacteria use shear force to trigger their attachment to discrete pockets in the host tissue. Autoaggregation of *E. amylovora* negatively affects its biofilm formation under static conditions, thus its biofilm formation in the plant xylem which leads to fireblight disease is hypothesized to be due to the shear force exerted by the movement of water up the xylem [42].

## Applications of autoaggregation studies

### Identification of therapeutic targets and alternatives

#### Colonization-inhibition therapies

Autoaggregation is one of many phenotypes that contribute to colonization and pathogenesis that is amenable to genetic and biochemical dissection. Better understanding of this phenomenon can inform alternative therapeutics to disrupt it [65, 66]. Inhibition of autoaggregation can be achieved by using compounds that compete for or modify the interaction site or that interfere with the assembly of autoaggregation factors. The assembly of the aggregative adherence fimbriae II, which is expressed by some enteroaggregative *E. coli* and confers autoaggregation [67], is inhibited by the antiparasitic agent nitazoxanide [68, 69]. Also, the disaggregation of the *N. meningitidis* aggregates can be achieved by the inhibition of the *N. meningitidis* PilF ATPase which leads type IV pilus disassembly[66].

#### Predicting probiotic potential

Probiotic organisms often protect against infection by autoaggregating or coaggregating with pathogens. Autoaggregation allows competitive exclusion and displacement of pathogens while coaggregation increases proximity of the co-aggregating probiotic bacteria's Type VI secretion systems and releases antimicrobial substance to the target pathogen [38]. The well-documented probiotic potential of *Lacticaseibacillus rhamnosus* GG lies in its immunogenic SpaCBA pili that mediate mucosal adherence and autoaggregation [70]. *L. rhamnosus* GG convinently outcompetes and inhibits *S. aureus* growth on keratinocytes; conversely, *L. rhamnosus* GG *spaCBA*, which had significantly reduced keratinocyte adhesion, autoaggregation and co-aggregation with *S. aureus*, favoured adherence and growth of *S. aureus* on kerationcytes [70].

### Identification of diagnostic potential

Organism-specific autoagglutinins eliciting visible autoaggregation can be utilized in disease diagnosis. Serum extracellular vesicles (EV) may mediate bacterial aggregation potentially specific enough to serve as a quick test to identify infection pathogens [71]. For example, serum-EVs from neutrophilic granulocytes/neutrophils isolated from osteomyelitis patients mediated aggregation of *S. aureus ex vivo*, albeit with weak cross-reaction with *P. aeruginosa*.

## Measuring autoaggregation *in vitro*

### Sedimentation in liquid medium

**Figure 4.**
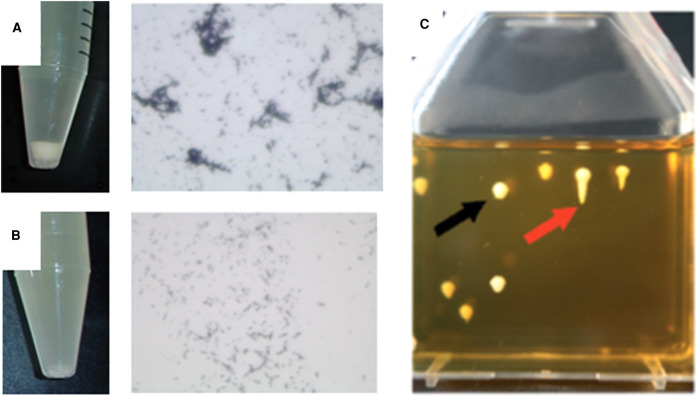
Visualizing autoaggregation as sedimentation. Autoaggregation in liquid media mediated by (**A**) the heat-resistant agglutinin 1 gene expressed in *Escherichia coli* from an arabinose inducible promoter on the pBAD vector after induction with arabinose (**B**) vector control prepared with the same protocol. The tubes show settling patterns in broth cultures left statically for 6 h and the photomicrographs are crystal violet stained mounts of cells taken from just above the pellet. (**C**) Autoaggregation chain-forming *Streptococcus salivarius* in semi-lquid media. Chain-forming streptococci produce spherical suspended colonies (black arrow) while mutants unable to form chains produce ‘roots’ in the semi-solid medium (red arrow). Reproduced from Couvigny et al. 2018 [31] under a Creative Commons Attribution License.

Bacterial aggregates settle faster and more compactly in liquid culture (Figure 4A,B) and this is the basis for the most common indirect method used to detect and quantify autoaggregation. Autoaggregation can be observed macroscopically as the floccules/sediments at the bottom of the static tube. Aggregate architecture can be evaluated by viewing a sample of the sediment by bright field, phase-contrast or fluorescent microscopy, depending on how the cells were labelled or stained (Figure 4C,D). The change in surface optical density over time can be used to compute autoaggregation rate. A detailed sample protocol optimized for *E. coli*, which can be adapted for other bacteria, is included as Supplementary Information.

### Flow cytometry

Flow cytometers use laser beam to sort cells in a milleu by size, complexity or content. The Forward Scatter Channel (FSC) signal is proportional to cell or aggregate size; the Side Scatter Chanel (SSC) Signal corresponds to structural complexity and granularity. A multiparametric analysis of both signals, with appropriate gating, will therefore sort and quantity single and aggregate cells in a sample. Aggregates will have higher FSC and SSC while single cells will have lower FSC and SSC [18, 21, 56].

### Sedimentation in semi-liquid medium

The viscosity of semi-liquid agar medium restricts the displacement of chain-forming *Streptococcus salivarius* and can be used to measure lateral autoaggregation. Trapped chains in extracellular agar-matrix yield round suspended colonies, while differentially displaced non-chain-forming bacteria escape agar-matrix immobilization resulting in colonies with faster sedimenting ‘roots’ (Figure 4C) [31].

### Atomic force microscopy

Atomic force microscopes (AFM) can be used to determine the force between the tip of a probe and the surface of a queried sample. The attractive force causes the cantilever attached to the tip to deflect and this deflection is detected most commonly by laser beam. The attractive force calculated can be used to determine the physicochemical and molecular properties of the sample. AFM has been used to probe the surface of cell aggregates to define their morphology and adhesive strength [21, 39].

## Perspectives

- Autoaggregation is a beneficial, complex and highly moderated bacterial phenomenon. It occurs via specific adhesive interactions and can be regulated transcriptionally, post-transcriptionally and epigenetically. Autoaggregation differentially correlates to adherence and biofilm depending on the assay conditions and phenotype-mediating factors.
- Autoaggregation is the result of submolecular interactions many of which remain to be deciphered. The complexities arising from autoaggregation require that the phenomenon be studied directly using multiple methods and evaluated in the context of more complex phenotypes to which it contributes.
- Identification of autoaggregating bacteria or factors will define new therapeutic targets and identify probiotic mechanisms, ultimately yielding alternatives to antimicrobials. Understanding autoaggregation is also key to unravelling bacterial colonization which is in turn fundamental to pathogen prevention, treatment and containment research.

## Abbreviations

AAS: African Academy of Sciences
AFM: atomic force microscopes
FSC: forward scatter channel
SAATs: self-associating autotransporters
SSC: side scatter chanel
