# Supplementary material for: Bacteria autoaggregation: how and why bacteria stick together
Source: Biochem Soc Trans. 2021 Jun 10;49(3):1147–57. doi: 10.1042/BST20200718 (PMC8286834; doi:10.1042/BST20200718)
Supplement: Supplementary Material [file BST-49-1147-s1.pdf]

Sedimentation in liquid medium:

1. **Preparing the organisms:** For fluorescence microscopy, test strains are labelled with fluorescent markers such as Green or red fluorescent proteins.
2. **Overnight growth:** Grow the test organisms overnight preferably with shaking.
3. **Determine the optical density of test organism(s):** Take the initial $OD600_{nm}$ reading of the overnight broth culture or making an emulsion of the test organism's colonies (1) to a predetermined $OD600_{nm}$.
   Modification
   OD adjustment step: This is achieved by diluting overnight culture (2–10). Dilution with a fresh medium can lead to an increase in growth and alter readings. Thus, after diluting with fresh medium, most setups involve agitated incubation to the early exponential phase of test bacterium (roughly OD600nm =0.25) before the transfer of test organisms into test tubes.
   Alternatively, OD can be adjusted by concentrating harvested, washed and resuspended cells from the overnight culture in non-growth medium eg PBS or pure water to a predetermined $OD600_{nm}$ (1,10–18).
   $OD600_{nm}$ levels are determined by researchers to have the same starting $OD600_{nm}$ for all organisms to be tested or for all replicates and are determined based on the growth kinetics of the test organism(s).
   Note of caution: Test organisms with curli should be prepared without centrifugation or vortexing to minimise curli detachment(19)
4. **Transfer of test organism(s):** Transfer a known volume (eg 10ml) of the test organisms into a round bottom test tube, a round-bottom microtitre well or a cuvette and incubated statically (20).
   Modification
   Two tube setup: Test organisms are transferred into two tubes –one labelled static the other vortex. Both are incubated statically. The control (vortex)tube will be vortexed before sample optical densities are measured. (4,6,8)
5. **Take time interval(s) absorbance reading(s):** After allowing to stand and at the predetermined time interval(s), the optical density of the sample is measured at the top or bottom of the tube/well.
   Top reading: A volume of the static culture is taken from the top of the medium into the cuvette (2) or microtitre plates (4,6). Volumes varies from 0.5ml (8) to 1ml (2) and 2mL (21) and are taken from 1cm (21) or 2cm from the top(8).
   For a one-tube set-up, after taking the static reading, vortex the tube and take final or vortexed OD reading. While for a two-tube set-up, the control tube is vortexed before sample is taken and OD determined(4,6,8).
   Bottom reading: Alternatively, bottom absorbance readings can be taken at predetermined time intervals throughout the experiments (22). This measures the increasing optical density at the bottom of the tube due to sedimenting cells; there is no vortexing.
6. **Image acquisition:** At the end of the experiment pictures of the test tubes are taken (macroscopic) (Figure 1a). For microscopic evaluation, the sediments at the bottom of

the tubes are viewed by taking a sample of the sediment at the bottom of the tube onto a glass slide or direct viewing of the titre well. Alternatively, sediments to be viewed can be placed on microscope slides coated with a thin agarose pad (3). A more accurate quantification can be achieved by the use of fixed-volume-gridded-well slides e.g. hemocytometers such that sample volume is fixed and counting is accurate (Figure 1b). Sediments can be viewed using different microscopes (bright field, Phase-contrast or fluorescence microscopy) depending on the fixing and staining methods applied (23,24). Acquired image can be qualitatively or quantitatively analysed using ImageJ (3,25–27). The aggregate diameter should be predetermined and guided by the size of the bacterium queried and autoaggregation can be expressed as the percentage of positive aggregates in total particles in the field. (27)

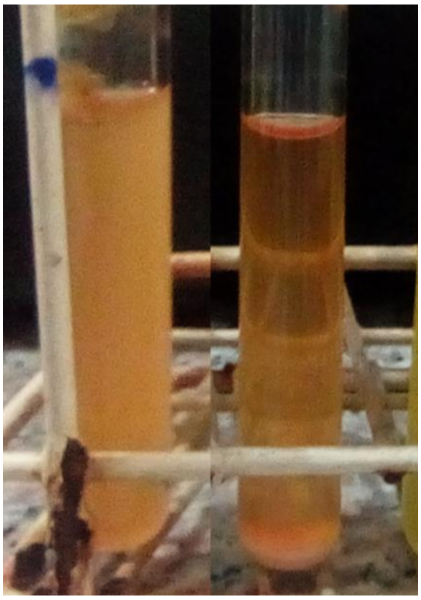

Suppl Figure 1 Paired ‘static and vortexted tubes late in an autoaggregation settling experiment

When microtitre plates are used the wells can be viewed using an inverted microscope and or round glass slips can be placed at the bottom of the well throughout the experiment. The glass slide can be removed and stained appropriately (5,26).

7. **Calculate the autoaggregation percentage:** Autoaggregation is calculated as the change in the initial or pre-vortexed optical density (28) (29).

$$100 \times \left(1 - \left[\frac{OD_{initial\ or\ before\ static\ incubation} - OD_{After\ incubation}}{OD_{\ initial\ or\ before\ incubation}}\right]\right) \text{ (30)}$$

Or

$$100 \times \left(1 - \left[\frac{OD_{\ After\ static\ incubation\ or\ pre-vortexed}}{OD_{\ initial\ or\ \ Vortexed}}\right]\right) \text{(1,4,6,7,9–14,31).}$$

If multiple readings (ideally at least five) were taken over time, plot autoaggregation curve. Additionally, the rate of sedimentation can be calculated by dividing autoaggregation calculated by time(32).

References

1. Couvigny B, Kulakauskas S, Pons N, Quinquis B, Abraham A-L, Meylheuc T, et al. Identification of New Factors Modulating Adhesion Abilities of the Pioneer Commensal Bacterium Streptococcus salivarius. Front Microbiol. 2018;9:273.
2. Ageorges V, Schiavone M, Jubelin G, Caccia N, Ruiz P, Chafsey I, et al. Differential homotypic and heterotypic interactions of antigen 43 (Ag43) variants in autotransporter-mediated bacterial autoaggregation. Sci Rep. 2019 Jul;9(1):11100.
3. Adams DW, Stutzmann S, Stoudmann C, Blokesch M. DNA-uptake pili of Vibrio cholerae are required for chitin colonization and capable of kin recognition via sequence-specific self-interaction. Nat Microbiol. 2019 Sep;4(9):1545–57.
4. Sorroche F, Bogino P, Russo DM, Zorreguieta A, Nievas F, Morales GM, et al. Cell Autoaggregation, Biofilm Formation, and Plant Attachment in a Sinorhizobium meliloti lpsB Mutant. Mol Plant Microbe Interact. 2018 Oct;31(10):1075–82.
5. Lang C, Fruth A, Holland G, Laue M, Mühlen S, Dersch P, et al. Novel type of pilus associated with a Shiga-toxigenic E. coli hybrid pathovar conveys aggregative adherence and bacterial virulence. Emerg Microbes Infect. 2018 Dec;7(1):203.
6. Primo E, Bogino P, Cossovich S, Foresto E, Nievas F, Giordano W. Exopolysaccharide II Is Relevant for the Survival of Sinorhizobium meliloti under Water Deficiency and Salinity Stress. Molecules. 2020;25(21).
7. Wang Y, Samaranayake LP, Dykes GA. Plant components affect bacterial biofilm development by altering their cell surface physicochemical properties: a predictability study using Actinomyces naeslundii. FEMS Microbiol Ecol. 2020 Oct;
8. Blanton L V, Wang LT, Hofmann J, Dubow J, Lafrance A, Kwak S, et al. Aggregative Adherence and Intestinal Colonization by Enteroaggregative Escherichia coli Are Produced by Interactions among Multiple Surface Factors. 2018;3(2):1–14.
9. Sterniša M, Klančnik A, Smole Možina S. Spoilage Pseudomonas biofilm with Escherichia coli protection in fish meat at 5 °C. J Sci Food Agric. 2019 Aug;99(10):4635–41.
10. Li D, Liang X, Wu C. Characteristics of Nitrogen Removal and Extracellular Polymeric Substances of a Novel Salt-Tolerant Denitrifying Bacterium, Pseudomonas sp. DN-23. Front Microbiol. 2020;11(March):1–12.
11. Petruzzi B, Dickerman A, Lahmers K, Scarratt WK, Inzana TJ. Polymicrobial Biofilm Interaction Between Histophilus somni and Pasteurella multocida. Front Microbiol. 2020;11:1561.
12. Bevilacqua A, Campaniello D, Speranza B, Racioppo A, Altieri C, Sinigaglia M, et al. Microencapsulation of Saccharomyces cerevisiae into Alginate Beads: A Focus on Functional Properties of Released Cells. Foods (Basel, Switzerland). 2020 Aug;9(8).
13. Klančnik A, Gobin I, Jeršek B, Možina SS, Vučković D, Žnidarič MT, et al. Adhesion of Campylobacter jejuni Is Increased in Association with Foodborne Bacteria. Microorganisms. 2020 Jan;8(2).

14. Miljkovic M, Marinkovic P, Novovic K, Jovcic B, Terzic-Vidojevic A, Kojic M. AggLr, a novel aggregation factor in Lactococcus raffinolactis BGTRK10-1: its role in surface adhesion. Biofouling. 2018 Jul;34(6):685–98.

15. Selvaraj A, Jayasree T, Valliammai A, Pandian SK. Myrtenol Attenuates MRSA Biofilm and Virulence by Suppressing sarA Expression Dynamism. Front Microbiol. 2019;10:2027.

16. Valliammai A, Selvaraj A, Sangeetha M, Sethupathy S, Pandian SK. 5-Dodecanolide inhibits biofilm formation and virulence of Streptococcus pyogenes by suppressing core regulons of virulence. Life Sci. 2020 Oct;262:118554.

17. Dong Y, Li S, Zhao D, Liu J, Ma S, Geng J, et al. IolR, a negative regulator of the myo-inositol metabolic pathway, inhibits cell autoaggregation and biofilm formation by downregulating RpmA in Aeromonas hydrophila. NPJ biofilms microbiomes. 2020 May;6(1):22.

18. Chen H-H, Chang C-C, Yuan Y-H, Liaw S-J. A CpxR-Regulated zapD Gene Involved in Biofilm Formation of Uropathogenic Proteus mirabilis. Infect Immun. 2020 Jun;88(7).

19. Cohen N, Zhou H, Hay AG, Radian A. Colloids and Surfaces B : Biointerfaces Curli production enhances clay- E . coli aggregation and sedimentation. Colloids Surfaces B Biointerfaces [Internet]. 2019;182(June):110361. Available from: https://doi.org/10.1016/j.colsurfb.2019.110361

20. Kharadi RR, Sundin GW. Physiological and Microscopic Characterization of Cyclic-di-GMP-Mediated Autoaggregation in Erwinia amylovora. Front Microbiol. 2019;10:468.

21. Zhang H, Wang Q, Liu H, Kong B, Chen Q. In vitro growth performance, antioxidant activity and cell surface physiological characteristics of Pediococcus pentosaceus R1 and Lactobacillus fermentum R6 stressed at different NaCl concentrations. Food Funct. 2020;11(7):6376–86.

22. Cohen N, Zhou H, Hay AG, Radian A. Curli production enhances clay-E. coli aggregation and sedimentation. Colloids Surf B Biointerfaces. 2019 Oct;182:110361.

23. Bunetel L, Tamanai-Shacoori Z, Martin B, Autier B, Guiller A, Bonnaure-Mallet M. Interactions between oral commensal Candida and oral bacterial communities in immunocompromised and healthy children. J Mycol Med. 2019 Sep;29(3):223–32.

24. Shetty D, Abrahante JE, Chekabab SM, Wu X, Korber DR, Vidovic S. Role of CpxR in Biofilm Development: Expression of Key Fimbrial, O-Antigen and Virulence Operons of Salmonella Enteritidis. Int J Mol Sci. 2019 Oct;20(20).

25. Demirdjian S, Sanchez H, Hopkins D, Berwin B. Motility-Independent Formation of Antibiotic-Tolerant Pseudomonas aeruginosa Aggregates. Appl Environ Microbiol. 2019 Jul;85(14).

26. Kennouche P, Charles-Orszag A, Nishiguchi D, Goussard S, Imhaus A-F, Dupré M, et al. Deep mutational scanning of the Neisseria meningitidis major pilin reveals the importance of pilus tip-mediated adhesion. EMBO J. 2019 Nov;38(22):e102145.

27. Deng S, Wang Y, Liu S, Chen T, Hu Y, Zhang G, et al. Extracellular Vesicles: A Potential

Biomarker for Quick Identification of Infectious Osteomyelitis. Front Cell Infect Microbiol. 2020;10:323.

28. Habouria H, Pokharel P, Maris S, Garénaux A, Bessaiah H, Houle S, et al. Three new serine-protease autotransporters of Enterobacteriaceae (SPATEs) from extra-intestinal pathogenic Escherichia coli and combined role of SPATEs for cytotoxicity and colonization of the mouse kidney. Virulence. 2019 Dec;10(1):568–87.

29. Béchon N, Jiménez-Fernández A, Witwinowski J, Bierque E, Taib N, Cokelaer T, et al. Autotransporters Drive Biofilm Formation and Autoaggregation in the Diderm Firmicute Veillonella parvula. J Bacteriol. 2020 Oct;202(21).

30. Spacova I, O'Neill C, Lebeer S. Lacticaseibacillus rhamnosus GG inhibits infection of human keratinocytes by Staphylococcus aureus through mechanisms involving cell surface molecules and pH reduction. Benef Microbes. 2020 Oct;1–14.

31. Wang Y, Lam ATW. Epigallocatechin gallate and gallic acid affect colonization of abiotic surfaces by oral bacteria. Arch Oral Biol. 2020 Oct;120:104922.

32. Abdel-Nour M, Su H, Duncan C, Li S, Raju D, Shamoun F, et al. Polymorphisms of a Collagen-Like Adhesin Contributes to Legionella pneumophila Adhesion, Biofilm Formation Capacity and Clinical Prevalence. Front Microbiol. 2019;10:604.
